# Evaluation of nurses' knowledge and performance regarding preparation and injection of intravenous drugs in pediatric wards in Iran

**DOI:** 10.1186/s12887-023-04336-z

**Published:** 2023-10-26

**Authors:** Amir Shahzeydi, Faramarz Kalhor, Sajjad Khaksar, Ali Mohammad Sabzghabaee, Fatemeh Joonbakhsh, Najmeh Ajoodanian

**Affiliations:** 1 Pediatric Cardiovascular Research Center, Cardiovascular Research Institute, Isfahan University of Medical Sciences, Isfahan, Iran; 2 Nursing and Midwifery Care Research Center, Faculty of Nursing and Midwifery, Isfahan University of Medical Sciences, Isfahan, Iran; 3 Trauma Center Care, Faculty of Nursing and Midwifery, Kashan University of Medical Science, Kashan, Iran; 4 Isfahan Clinical Toxicology Research Center, Isfahan University of Medical Sciences, Isfahan, Iran; 5 Faculty Member of Pediatrics Nursing, School of Nursing and Midwifery, Isfahan University of Medical, Isfahan, Iran

**Keywords:** Nurse, Knowledge, Performance, Infusions, Intravenous, Child

## Abstract

**Background:**

A error in intravenous injection in pediatric wards can cause irreparable injuries. This study aimed to determine the level of knowledge and performance of nurses in terms of preparation and injection of intravenous drugs in pediatric wards of hospitals affiliated to Isfahan University of Medical Sciences.

**Methods:**

This cross-sectional study was conducted in 2022 on 156 nurses working in pediatric wards. The data was collected with demographic information questionnaire and the knowledge and performance of the participants were determined using a researcher-made questionnaire, including the five rights of medication administration (preparation and injection, medication error, drug side effects, family empowerment, and documentation) using self-reporting and observation methods. Formal and content validity was calculated using the opinions of 10 experts and Cronbach's alpha with 40 samples.

**Results:**

The mean and standard deviation of total nurses' knowledge and performance scores were 58.31 + 10.1 and 66.1 + 14.4, respectively. Moreover, the mean and standard deviation of nurses' knowledge scores were 63.55 + 14.3 for documentation, 46.1 + 7.9 for preparation and injection, 73.9 + 12.3 for drug side effects, 58.4 + 10.2 for medication error, and 69.4 + 9.4 for family empowerment. Besides, the mean performance was 69.1 + 17.6 for documentation, 61.3 ± 9.9 for preparation and injection, 78.21 + 12 for drug side effects, 58.6 + 15 for medication error, and 65.4 + 17.7 for family empowerment.

**Conclusion:**

The results showed that the mean knowledge and pharmacological performance of nurses working in pediatric wards in different areas of the principles of medicine were not at the desired level, and this can affect children adversely.

## Background

The health care system in Iran has moved towards implementing clinical governance like other health care systems at the international level since 2009 to ensure that health care is provided to members of the community with the highest possible standards of care and guarantees patient safety [1]. In a system based on clinical governance, patients and, in fact, clients of the health care system are at the heart of the care process, so efforts are made to provide the community with high-quality services according to the desired principles to improve work standards [2]. The health system based on clinical governance has seven important axes (including patient and community participation, information use, education and learning, clinical effectiveness, clinical audit, and risk management). In the field of risk management, medication errors are a major concern and a global issue related to patient care safety [3].

Types of medication errors include prescription errors, drug injection errors, distribution errors, and patient compliance errors, which can occur due to the lack of experience or knowledge about the drug, failure to use the basic rules, misspellings of the drug name, and ignorance of important information such as the patient's allergies [1].

The National Health Service (NHS) of England estimates that medication errors account for about 20% of drug-related deaths [4]. It can also be claimed that 61% of life-threatening errors are related to intravenous drugs [5, 6]. Studies in the United Kingdom and the United States confirm that nurses make 13% -84% mistakes in the preparation and use of intravenous drugs annually [7]. However, cytotoxic drugs, Total Parenteral Nutrition (TPN), and drugs needed to relieve acute pain are provided separately by pharmacists [8]. However, in many countries, such as Iran, the preparation and injection of intravenous drugs is the responsibility of nurses [9].

In some studies, many medication errors have been associated with the lack of medication knowledge, the lack of appropriate skills in prescribing medication [10, 11]. Besides, other studies have mentioned failure to communicate and follow clinical policies and guidelines as the main causes of medication errors in addition to the lack of knowledge and skills [12, 13]. Low quality and errors in the preparation and administration of intravenous drugs among health professionals are strategic issues that reduce the quality of health care in general [14]. Moreover, it can lead to adverse consequences, such as endangering patients' health, increasing health care costs, and even death. However, children are more at risk of medication errors than adults. Because the ability to communicate is limited in children, especially younger ages. Further, the lack of sufficient information for the preparation and injection of medicine for children by the drug manufacturer can lead to medication errors [15]. Therefore, the quality of this process is crucial in providing safe patient care, and there must be continuous efforts to improve it [16]. Regarding the safe application of intravenous drugs, health care providers need to have sufficient knowledge and skills, while continuous participation in training programs can increase the knowledge and awareness of nurses [17, 18]. There is no comprehensive pediatric study that reflects the current situation to be used for quality improvement planning. Therefore, this study aimed to determine the level of knowledge and performance of nurses in terms of preparation and injection of intravenous drugs in pediatric wards of hospitals affiliated to Isfahan University of Medical Sciences.

## Methods

### Design and participants

This cross-sectional study was performed over a 6-month period in the pediatric wards of hospitals affiliated to Isfahan University of Medical Sciences. To conduct the study, the hospitals affiliated to Isfahan University of Medical Sciences were referred Sampling was performed by census method among individuals with inclusion criteria. Overall, 300 nurses were working in the study wards, of whom 160 met the inclusion criteria. Inclusion criteria were at least 6 months of work experience in pediatric wards regardless of status or shift, working in pediatric emergency departments, pediatric internal medicine, intensive care and pediatric surgeryExclusion criteria were unwillingness to participate in the study, absence from the hospital during the study period due to annual leave or childbirth, and a distorted and invalid questionnaire.

### Data collections

A questionnaire of pharmacological knowledge was distributed among the participants at the beginning of the morning, evening and night shift after obtaining writing consent, by a member of the research team, and the nurses had the opportunity to answer the questionnaires until the end of the shift. Questionnaires were collected at the end of the shift, leading to 156 valid and 4 invalid questionnaires. The performance of nurses was observed using the observation method, which was carried out in three medication situations to complete the observation checklist of performance and the average of three observations as the overall performance score.

### Research tools

The study tool comprised three parts: the first part of the questionnaire included demographic information, such as gender, education, age, ward, work experience, type of employment, job satisfaction and working hours per month, which were completed by self-report by nurses.

In order to prepare the questionnaire, the ward nurses were first asked to raise questions in five areas of documentation, drug preparation, drug injection, drug side effects, and medication error to evaluate their staff drug knowledge. Then, their opinions were collected and questions were reviewed and categorized with scientific sources [19, 20]. Formal and content validity was determined using the opinions of 10 experts (three pediatric nursing professors, one clinical pharmacist, two pediatric medical professors, and four senior nursing experts) (CVI = 0.8 and CVR = 0.8). Cronbach's alpha was performed with 40 samples (0.735) for instrument reliability, while HTMT was also calculated for divergent validity of the structure (0.4).

The pharmacological Knowledge Questionnaire consisted of 20 questions classified into five areas of preparation and injection (nine statements), family empowerment (four statements), documentation (two statements), drug side effects (three statements), and medication error (two statements). The answers to the questions were in the form of a four-point Likert scale (correct, I do not know, and 2 false answers). The scores ranged from 0 to100 for ease of data analysis. The total score of the questionnaire was defined as good, acceptable, poor, and unacceptable for scores of > 90, 75–90, 50–75, and < 50, respectively. These statements had quantitative nature.

A 20-item checklist was used to evaluate pharmacological performance. This checklist consists of five areas of preparation and injection (nine statements), family empowerment (four statements), documentation (two statements), drug side effects (three statements), and medication error (two statements). The performance was observed by a research team member and completed the three-point Likert scale (It is done right, it is done in completely, it is done wrong). The checklist reliability was measured by the agreement coefficient of two observers in 10 drug positions (*r* = 0.8). The performance of each nurse was observed in three situations and the average of three observations was recorded as the performance of each person. The scores ranged from 0 to100 for ease of data analysis, respectively.

### Statistical analyze

The collected data were statistically analyzed by SPSS VER 16 software by using KS, independent t-test, Chi-square, One-Way ANOVA, Pearson and Spearman. Furthermore, the open-ended questions were considered to gain a general understanding of the nurses' point of view. After summarizing the descriptive answers, they were examined for accuracy and analyzed using content analysis.

## Results

A total of 160 questionnaires were distributed among nurses, 156 of which were accepted for the study. Women made up 96% of study participants. The mean age of nurses participating in the study was 32.29 + 4.09 years old, and the average work experience of staff was 12.6 + 3.12. Regarding the level of education, 88% of the participants had a bachelor's degree, and 12% had a master's degree in nursing. Job satisfaction among nurses was on average 68.5%, but no significant difference was observed between nurses working in pediatric wards.

**Table:**
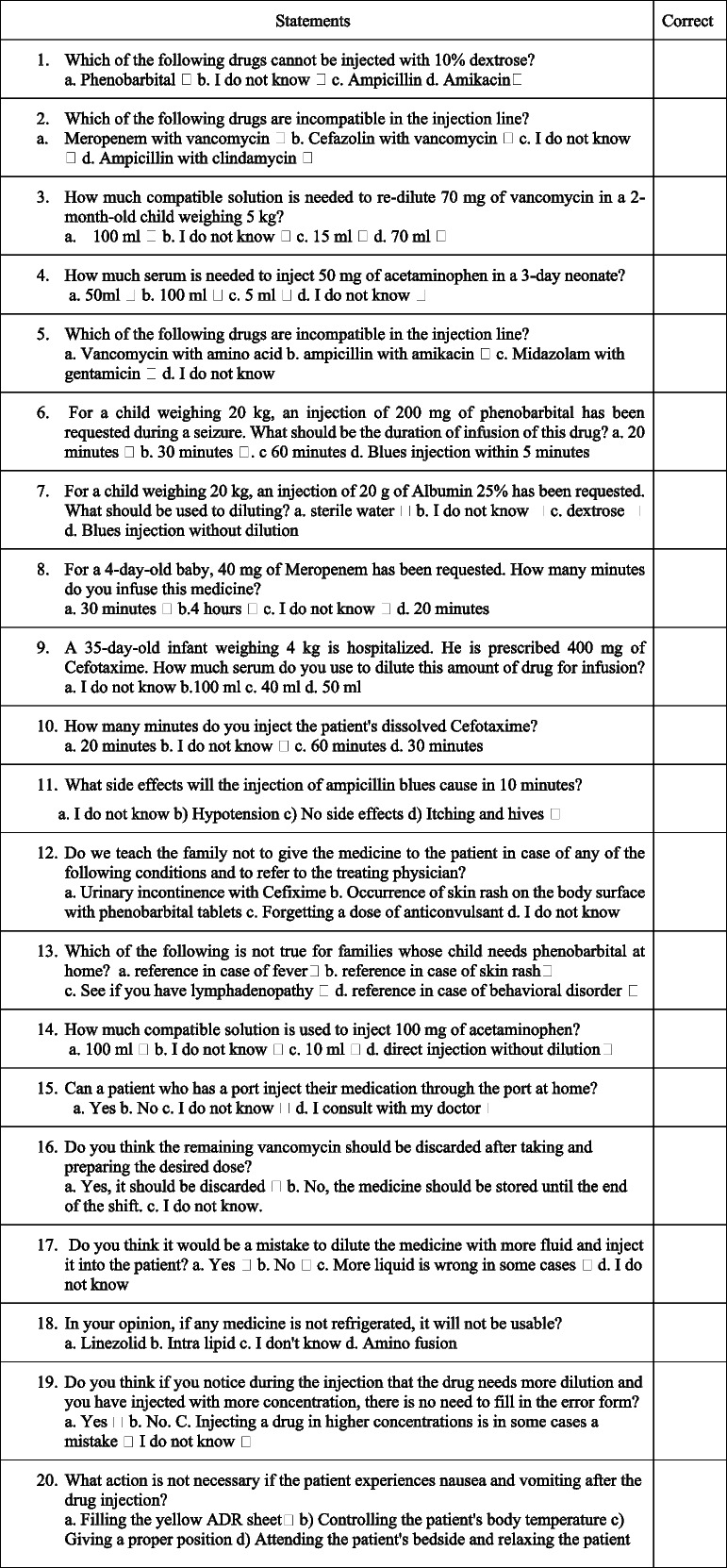

The mean and standard deviation of total nurses' knowledge scores were 58.31 + 10.1, respectively. Additionally, the mean and standard deviation of nurses' knowledge scores were 63.55 + 14.3 for documentation, 46.1 + 7.9 for preparation and injection, 73.9 + 12.3 for drug side effects, 58.4 + 10.2 for medication error, and 69.4 + 9.4 for family empowerment. The mean score of pharmacological knowledge of different pediatric wards examined in this study included pediatric surgery ward (62.31 + 7.52), pediatric internal ward (57.31 + 3.31), pediatric emergency department (60.97 + 4.74), and pediatric intensive care unit (56.67 + 3.62). There was a significant difference in the analysis of the results (*p* < 0.05). Table 1 shows the results of the statistical analysis of data in different areas of pharmacological knowledge.

**Table 1:** Comparison of the average score of nurses' pharmacological knowledge in different areas of pharmacological knowledge

Scope of pharmacological knowledge		Internal	Surgery	Emergency	PICU	
						P*
		Mean (SD)				
Preparation and injection	Drug compatibility	43.6(6.9)	40.1(8.2)	53.8(8.6)	48.8(9.2)	.079
	Storage of prepared drugs	50.0(7.9)	35.2(7.3)	32.3(8.2)	24.4(8.2)	.000
	dilution of drugs	53.8(5.8)	40.7(6.9)	48.4(8.8)	52.9(9.1)	.115
	Duration of injection	44.9(6.4)	51.8(9.5)	69.9(8.7)	48.9(7.2)	.000
Medication Error Knowledge	Medication error report	44.5(12.3)	38.4(8.1)	50.0(8.6)	41.1(11.6)	0.196
	Medication error detection	80.1(10.2)	76.2(11.6)	64.9(9.2)	72.2(10.3)	0.006
Knowledge of drug side effects	Identification of drug side effects	76.9(11.2)	75.8(12.6)	82.7(13.2)	72.8(12.4)	.275
	Report of drug side effects	78.9(12)	70.8(11.3)	60.8(11.6)	72.2(14.3)	.275
Family empowerment	Knowledge of home care	77.9(9)	79.6(8.2)	64.5(10.8)	55.6(9.4)	.013
Documentation		60.1(12.2)	66.1(18)	65(15.3)	63(11.7)	0.23
Mean of total knowledge		61.1(9.4)	57.5(10.2)	59.3(10.3)	55.2(10.3)	0.000

^*^One-way ANOVA

Accordingly, as shown in Table 2, only 16.1% of emergency department nurses, 34.6% of pediatric surgical nurses, 6.7% of intensive care unit nurses and 13% of pediatric intensive care unit nurses were within an acceptable range, and the rest of them (84.5%) got a lower-than-expected knowledge score (poor and unacceptable) (Table 2).

**Table 2:** Comparison of knowledge levels in different pediatric wards

		Knowledge levels				
		Unacceptable (< 50)	Poor (50–75)	Acceptable (75 -90)	Good (> 90)	
Pediatric surgery	Frequency	9	8	8	1	p*
	%	34.6%	30.8%	30.8%	3.8%	.000
Pediatrics internal	Frequency	11	36	6	1	
	%	20.4%	66.7%	11.1%	1.9%	
Pediatric Emergency	Frequency	3	23	5	0	
	%	9.7%	74.2%	16.1%	0.0%	
PICU	Frequency	11	31	3	0	
	%	24.4%	68.9%	6.7%	0.0%	
Total	Frequency	34	98	22	2	
	%	21.7%	62.8%	14.1%	1.2%	

^*^Kruskal Wallis

The mean and standard deviation of total nurses' performance scores was 66.1 + 14.4, respectively. In addition, the mean performance was 69.1 + 17.6 for documentation, 61.3 ± 9.9 for preparation and injection, 78.21 + 12 for drug side effects, 58.6 + 15 for medication error, and 65.4 + 17.7 for family empowerment. Analysis of statistical results showed that the performance of nurses in different areas of drug administration was significantly different (*P* > 0.001). The best performance among nurses in the pediatric internal ward had a mean (SD) of 71 (14.3), and the worst performance was related to the pediatric surgical ward with a mean (SD) of 62 (12.9) (Table 3).

**Table 3:** Comparison of the average performance of nurses in different areas of medicine in different clinical wards

Scope of pharmacological performance		Internal pediatrics	Pediatric surgery	Emergency	PICU	
						P*
		Mean (SD)				
Preparation and injection	Drug compatibility	62(11.7)	49.3(13.9)	61.3(14.3)	48.8(12.9)	.003
	Storage of prepared drugs	54.1(16.5)	35.2(12.3)	42.3(16.2)	53.4(12.6)	0.004
	dilution of drugs	72.2(15.6)	61.5(11.1)	77.2(13.9)	64.4(18.1)	.0160
	Duration of injection	79.6(12.8)	69.3(11.1)	67.4(17.9)	82.3(8.5)	.0820
Medication Error	Prevention of drug microbial resistance	48.2(16.1)	45.9(13.1)	80.81(18.6)	42.2(13.2)	.0050
	Medication error prevention	79.6(11.9)	75.9(12.8)	83.8(17.9)	82.2(18.6)	.4080
	Drug preparation	41.3(16.9)	38.2(13.9)	48.6(14.6)	36.3(12.3)	.2900
Drug side effects	Identification of drug side effects	86.9(10.1)	78.78(2.6)	75.28(9.6)	86.6(16.2)	.3330
	Report of drug side effects	33.9(12.3)	30.7(16.1)	48.8(15.3)	32.2(13.9)	0.001
Family empowerment	Education and family empowerment	74.1(7.4)	62(29.4)	58(25.5)	67.6(8.6)	.340
Documentation		78(26.1)	73(15.1)	61.3(13.1)	64.1(16.2)	0.001
Mean of total performance		71(14.3)	62(12.9)	66.3(16.1)	65(8.1)	0.001

^*^One-Way ANOVA

Analysis of the findings showed a weak and direct correlation between drug knowledge and performance (*p* < 0.05, *r *= 0.128). Besides, there was a significant correlation between shift work and knowledge (*r* = 0.325) and drug performance (*r* = 0.248). Moreover, there was a weak correlation between job satisfaction and drug knowledge (*r* = 0.163). Contrary to expectations, the observations showed an inverse correlation between work experience and knowledge (*r* = -0.306) and drug performance (*r* = -0.232) (Table 4).

**Table 4:** Assessing the relationship between nurses' mean performance score and medication knowledge score, job satisfaction, working hours per month, education, work experience

		Work Experience	Job Satisfaction	Working hours per month	Mean pharmacological Knowledge Score
Job satisfaction	Correlation	.074	1		
	p	.217			
	N	156	156		
Working hours per month	Correlation	-.788**	-.029	1	
	p	.000	.635		
	N	156	156	156	
Mean pharmacological knowledge Score	Correlation	-.306**	.163**	.325**	1
	p	.000	.007	.000	
	N	156	156	156	156
Mean performance score	Correlation	-.232**	.055	.248**	.128*
	p	.000	.361	.000	.027
	N	156	156	156	156

*Spearman

**Pearson correlation

## Discussion

The aim of this study was to determine the knowledge and performance of nurses regarding the preparation and injection of intravenous drugs. According to the results of the present study, 84.5% of the participants in the study had a lower-than-expected knowledge (poor and unacceptable) in this test, indicating insufficient knowledge. Ashtiani et al. examined the pharmacological knowledge of heart hospital nurses using a questionnaire of nurses 'pharmacological knowledge in the fields of drug mechanism, drug side effects and nursing care. The results showed that nurses' knowledge was at an average level [21]. Unlike the present study, which examined only the pharmacological knowledge of pediatric public hospital nurses, they also examined the pharmacological knowledge between public and private nurses, which, although did not show a significant difference, in future studies, it seems that more attention will be paid to private sector nurses in this field.

Moreover, Salehifar et al. surveyed nurses in the field of knowledge, attitude and practice about the side effects of drugs and their reporting with a standard questionnaire of the Pharmacovigilance Research Group in Europe. The results of their study showed that most participants do not have sufficient knowledge in the field of drug monitoring and often provide incomplete definitions. Therefore, it seems that nurses' pharmacological knowledge in various fields suffers from several shortcomings [22]. Therefore, it can be acknowledged that measures to improve nurses' knowledge should be one of the important goals in healthcare because the results of several studies in different geographical locations in the country show weak to moderate medical knowledge of nurses.

Another study conducted in South India to examine the knowledge, attitude and practice of nurses' pharmacovigilance towards the reporting of drug side effects showed that most participants showed good knowledge and knowledge in this field. However, the transfer of knowledge to practice was not enough [23].

A study conducted at a university hospital in Rome to assess the knowledge, educational needs, behavior and attitudes of nurses about medication errors and the steps of intravenous drug administration showed that the samples had good knowledge, positive attitude and correct behavior related to the preparation and administration of intravenous drugs [24].

The study of studies abroad and at the home of the study of pharmacological knowledge of nurses at different levels showed that it seems that in order to improve the quality of health care and ensure the safety of patients, more investment should be made in training programs for nurses and promoting their knowledge and adequate knowledge of medical errors and prevention of special tools.

In addition to the above, only 7% of the participants had good pharmacological knowledge. In the study conducted by Arshadi et al., the level of knowledge of nurses was moderate. The reason for the difference in the results of these two studies could be the sampling context because Arshadi et al. studied the neonatal intensive care unit. The results of the study conducted by Khajeh Ali et al. showed that approximately 50% of nurses working in intensive care units and 33% of nurses in general wards were good at recognizing and using drugs and medication computing skills, which is not consistent with the present study probably due to the number of nurses participating in that study, the questionnaire used, etc.

The results of the present study showed that only 16.1% of emergency department nurses, 34.6% of pediatric surgical nurses, 6.7% of intensive care unit nurses, and 13% of pediatric intensive care unit nurses had an acceptable and good response, and the rest had lower knowledge scores (weak and unacceptable). Contrary to the present study, Ashtiani et al. and Khajeh Ali et al. reported higher drug knowledge scores for the nurses in the intensive care unit compared to those in general wards [21, 25]. This difference in the results may be due to the different number or work experience of nurses in these two studies.

According to the results, the nurses had weak or even unacceptable knowledge about the preparation and injection of drugs in all wards. Ashtiani et al. showed that nurses had moderate knowledge about drug side effects and nurse care in the field of pharmacological mechanisms; however, they had good knowledge about the preparation and injection of drugs. Therefore, there is a need to train nurses in the field of pharmacological retraining and especially how to prepare and inject drugs.

In addition, the findings of this study demonstrated a significant relationship between job satisfaction and the number of shifts and a significant inverse relationship between work experience and performance and medication knowledge. The results of studies conducted by Ashtiani regarding experience and shift work and drug knowledge of nurses and the study performed by Haji Babaei showed no significant differences between age and medication errors [21, 26]. Studies by Ito et al. and Sheu et al. have revealed that work experience further reduces medication errors [27, 28].

This indicates that the accuracy of medication calculations and drug knowledge decreases with the increase in nurses' experience, which is probably because newly graduated nurses remember much information while this is not true about experienced nurses, necessitating in-service training [25].

Considering that medication errors have become one of the most significant problems affecting patient safety in hospitals today, and given the large percentage of people with low drug knowledge in pediatric wards as shown in the present study, those involved in education in hospitals and the officials of the relevant committees are expected to provide continuous training courses and familiarize clinical staff with pharmacovigilance. Further, it is necessary to provide the necessary facilities for nurses to access information about drug complications and interactions while providing access to clinical pharmacologists in pediatric wards as well. Besides, based on the findings and the high sensitivity of working with children, it is recommended to use nurses with the necessary pharmacological knowledge in the workforce to prevent the occurrence of drug side effects in pediatric wards.

## Conclution

Overall, the nurses in this study had a moderate and below-average level of knowledge in the preparation and administration of intravenous drugs. Besides, their performance in different areas of medicine was at an average and below-average level. In addition, this study showed that the experience of nurses had no effects on the level of knowledge of these nurses in the preparation and injection of drugs and was only helpful in identifying the side effects of drugs.

Nurses' training should place more emphasis on these skills, and nursing education programs should provide more hours to calculate and understand the various areas of intravenous methods and drug safety issues. The participation of clinical pharmacists in nursing education programs in various areas related to intravenous drugs will be beneficial.

### Limitation

This study had some limitations, and the results may not necessarily reflect the knowledge status of all nurses working in pediatric wards. Knowledge measurement questions (only 20 items in the questionnaire) were very few and did not cover different aspects of injectable drugs such as drug interactions, pre-, intra-, and post-injection care and drug reaction treatment.The presence of an observer in the research field and observing the behavior of nurses could lead to the Hawthorne effect, which was tried to reduce with a long-term presence.

## Data Availability

The data were presented in tables.

## References

[1] Ravaghi H, Rafiei S, Heidarpour P, Mohseni M (2014). Facilitators and barriers to implementing clinical governance: a qualitative study among senior managers in Iran. Iran J Public Health.

[2] Donaldson L. Clinical governance: a quality concept. In: Tim Van Zwanenberg JH, editor. Clinical governance in primary care. 2nd ed. CRC Press; 2016. p. 1–16.

[3] Alghamdi AA, Keers RN, Sutherland A, Ashcroft DM (2019). Prevalence and nature of medication errors and preventable adverse drug events in paediatric and neonatal intensive care settings: a systematic review. Drug Saf.

[4] Sutherland A, Canobbio M, Clarke J, Randall M, Skelland T, Weston E (2020). Incidence and prevalence of intravenous medication errors in the UK: a systematic review. Eur J Hosp Pharm.

[5] Alrabadi N, Haddad R, Haddad R, Shawagfeh S, Mukatash T, Al-rabadi D, Abuhammad S (2020). Medication errors among registered nurses in Jordan. J Pharm Health Serv Res.

[6] Luokkamäki S, Härkänen M, Saano S, Vehviläinen-Julkunen K (2021). Registered Nurses' medication administration skills: a systematic review. Scand j caring sci.

[7] Sutherland A, Phipps DL, Tomlin S, Ashcroft DM (2019). Mapping the prevalence and nature of drug related problems among hospitalised children in the United Kingdom: a systematic review. BMC pediatr.

[8] Shitu Z, Aung MMT, Tuan Kamauzaman TH, Ab Rahman AF (2020). Prevalence and characteristics of medication errors at an emergency department of a teaching hospital in Malaysia. BMC health serv res.

[9] Abbasinazari M, Zareh-Toranposhti S, Hassani A, Sistanizad M, Azizian H, Panahi Y (2012). The effect of information provision on reduction of errors in intravenous drug preparation and administration by nurses in ICU and surgical wards. Acta Medica Iranica.

[10] Iqbal MS, Iqbal MZ, Rajan S, Ahmed NJ (2020). Evaluation of drug-related knowledge and clinical skills among future healthcare professionals. J Pharmaceutical Res Int..

[11] Al Meslamani AZ (2019). Investigating medication prescribing errors in the emergency department of a governmental hospital and measuring pharmacy students' knowledge and attitude of prescribing errors in Jordan.

[12] Thomas B, Paudyal V, MacLure K, Pallivalapila A, McLay J, El Kassem W, Al Hail M, Stewart D (2019). Medication errors in hospitals in the Middle East: a systematic review of prevalence, nature, severity and contributory factors. Eur J Clin Pharmacol.

[13] Musharyanti L, Claramita M, Haryanti F, Dwiprahasto I (2019). Why do nursing students make medication errors? A qualitative study in Indonesia. J Taibah Univ Med Sci.

[14] Strbova P, Mackova S, Miksova Z, Urbanek K (2015). Medication errors in intravenous drug preparation and administration: a brief review. J Nurs Care.

[15] Manias E, Cranswick N, Newall F, Rosenfeld E, Weiner C, Williams A, Wong IC, Borrott N, Lai J, Kinney S (2019). Medication error trends and effects of person-related, environment-related and communication-related factors on medication errors in a paediatric hospital. J paediatr child health.

[16] Mohiuddin AK (2020). The Role of the Pharmacist in Patient Care: Achieving High Quality, Cost-Effective and Accessible Healthcare through a Team-Based.

[17] Di Muzio M, De Vito C, Tartaglini D, Villari P (2017). Knowledge, behaviours, training and attitudes of nurses during preparation and administration of intravenous medications in intensive care units (ICU). A multicenter Italian study. Appl Nurs Res.

[18] Di Simone E, Tartaglini D, Fiorini S, Petriglieri S, Plocco C, Di Muzio M (2016). Medication errors in intensive care units: nurses’ training needs. Emerge Nurse.

[19] Phelps SJ, Hagemann TM, Lee KR, Thompson AJ. Pediatric injectable drugs: the Teddy bear book. 11th ed. Ashp; 2016. p. 944.

[20] Young T, Magnum B: Micormedex NeoFax Essentials; 2020:1(24)

[21] Ashtiani F, Hadavand N, Momeni B, Ansarifar A (2019). Evaluation of knowledge of pharmacology among nurses at Rajaie heart hospital and its position in care ethics. Iran J Biomed Law Ethics.

[22] Salehifar E, Ala S, Gholami K (2007). Knowledge, attitude and performance of pharmacists and nurses in Mazandaran province, Iran regarding adverse drug reaction and its reporting, 2005. Journal of Mazandaran University of Medical Sciences.

[23] Ganesan S, Vikneswaran G, Reddy KC, Subrahmanyam D, Adithan C (2016). A Survey on Knowledge, Attitude and Practice of Pharmacovigilance towards Adverse drug reactions reporting among Doctors and Nurses in a Tertiary Care Hospital in South India. Journal of Young Pharmacists.

[24] Di Simone E, Giannetta N, Auddino F, Cicotto A, Grilli D, Di Muzio M (2018). Medication errors in the emergency department: knowledge, attitude, behavior, and training needs of nurses. Indian J Crit Care Med.

[25] Khajeali N, Baghaei R (2014). Comparison of pharmacological knowledge and skills in pharmaceutical calculations in nurses of general and ICU wards in educational hospitals in Ahvaz. QJ Nurs Manage.

[26] Hajibabaee F, Joolaee S, Peyravi H, Haghani H. The relationship of medication errors among nurses with some organizational and demographic characteristics. Iran J Nurs Res. 2011;6(20):83–92.

[27] Ito H, Yamazumi S (2003). Common types of medication errors on long-term psychiatric care units. Int J qual health care.

[28] Sheu SJ, Wei IL, Chen CH, Yu S, Tang FI (2009). Using snowball sampling method with nurses to understand medication administration errors. J clin nurs.

